# Examining distinct working memory processes in children and adolescents using fMRI: Results and validation of a modified Brown-Peterson paradigm

**DOI:** 10.1371/journal.pone.0179959

**Published:** 2017-07-13

**Authors:** Vanessa Siffredi, Pierre Barrouillet, Megan Spencer-Smith, Maarten Vaessen, Vicki Anderson, Patrik Vuilleumier

**Affiliations:** 1 University of Geneva, Geneva, Switzerland; 2 Murdoch Childrens Research Institute, Melbourne, Australia; 3 University of Melbourne, Melbourne, Australia; 4 Monash University, Melbourne, Australia; 5 Royal Children’s Hospital, Melbourne, Australia; Chinese Academy of Sciences, CHINA

## Abstract

Verbal working memory (WM) comprises different processes (encoding, maintenance, retrieval) that are often compromised in brain diseases, but their neural correlates have not yet been examined in childhood and adolescence. To probe WM processes and associated neural correlates in developmental samples, and obtain comparable effects across different ages and populations, we designed an adapted Brown-Peterson task (verbal encoding and retrieval combined with verbal and visual concurrent tasks during maintenance) to implement during functional magnetic resonance imaging (fMRI). In a sample of typically developing children and adolescents (n = 16), aged 8 to 16 years, our paradigm successfully identified distinct patterns of activation for encoding, maintenance, and retrieval. While encoding activated perceptual systems in posterior and ventral visual regions, retrieval activated fronto-parietal regions associated with executive control and attention. We found a different impact of verbal versus visual concurrent processing during WM maintenance: at retrieval, the former condition evoked greater activations in visual cortex, as opposed to selective involvement of language-related areas in left temporal cortex in the latter condition. These results are in accord with WM models, suggesting greater competition for processing resources when retrieval follows within-domain compared with cross-domain interference. This pattern was found regardless of age. Our study provides a novel paradigm to investigate distinct WM brain systems with reliable results across a wide age range in developmental populations, and suitable for participants with different WM capacities.

## Introduction

The ability to maintain relevant information in mind in the presence of interference or distracting information is critical for higher cognitive functions required in daily life. Working memory (WM) is the theoretical construct used to refer to this capacity to simultaneously maintain and process information over brief periods of time according to current task goals [1–3]. Studies in children and adolescents show that WM capacity plays a crucial role in the development of many cognitive activities (e.g., learning, reasoning, problem solving, language comprehension), and also predicts academic performance and achievement [4–6]. Moreover, WM is impaired in various developmental disorders, e.g. attention deficit hyperactivity disorder (ADHD), autism spectrum disorder (ASD) or specific language impairment (SLI), providing a crucial neuropsychological measure in several neuropsychiatric conditions and useful risk marker for cognitive development [7–9].

From a developmental point of view, WM capacity develops rapidly over childhood [10–13]. This is usually measured by the increase in the amount of information that can be retained and transformed using complex memory span tasks that require maintaining information for further recall while performing a concurrent activity [7]. An important component of WM maintenance, involving active verbal rehearsal and attentional refreshing, emerges around 7 years of age [14]. Evidence suggests that multiple mechanisms contribute to childhood development of WM, affecting all the processes involved in encoding, maintenance, and retrieval (e.g., increase in attentional capacity, process automatisation, increase in knowledge, mnemonic strategies, and so forth; see [15]).

In terms of neural substrates, development of WM ability parallels structural changes in frontal-parietal cortices affecting grey matter [16] and white matter [17]. Similar to neuroimaging findings in adult populations, this core network of fronto-parietal brain areas is consistently found to activate in children and adolescents, and is apparent as early as 5 years of age during different verbal and visuospatial tasks thought to evaluate WM functions [18–20]. One recent imaging study compared encoding and retrieval processes in a Sternberg item recognition paradigm with digits in children and adolescents from 9 to 19 years [21]. Encoding of digits activated the right prefrontal and parietal cortex, left motor areas, occipital cortex, and cerebellum; retrieval activated the left prefrontal and parietal cortex, right motor areas, as well as anterior and posterior cingulate cortex, and cerebellum. Other functional neuroimaging studies investigating WM in school-age children have used an n-back task in which a sequence of stimuli is presented to the participant who must indicate when the current stimulus matches the one from *n* steps earlier in the sequence (e.g., [22, 23]). Despite its popularity in fMRI studies, empirical evidence shows that the n-back task correlates weakly with WM span tasks, suggesting that it is unlikely that these two types of tasks reflect a single construct, and questionning the empirical validity of using n-back tasks (continuous-recognition or updating measures) as a WM task [24, 25]. Other tasks, such as the Steinberg item recognition paradigm (e.g., [12, 26]), have also been used to study WM in developmental populations. However, these tasks require the maintenance of information in short-term memory, but not the simulatenous maintenance and manipulation of information as the theorethical construct of WM specifies [3, 27]. Thus, very few developmental studies have explored the neural correlates of WM using tasks requiring not just maintenance, but also active manipulation of information [18, 19]. To our knowledge, brain activity associated with WM processes of maintenance during the simultaneous processing of a concurrent task and retrieval have not yet been studied in developmental fMRI studies.

Previous literature has identified the major challenges inherent in studying both typical and atypical development, including designing tasks that can be administered to individuals across a wide age range in both typical and atypically developing groups [28]. In this study, our aims were to design a novel WM paradigm that: i) is demanding of WM capacity but simple enough to be administered to both children and adolescents and both healthy and clinical paediatric populations (e.g., populations with mild intellectual difficulties), and for which brain activity could not be explained by difference in age or WM performance; ii) would enable investigation of neural substrates for encoding, maintenance and retrieval WM processes during fMRI; and could identify the effect of different concurrent processing tasks on maintenance and retrieval.

Among the paradigms appropriate for measuring the impact of concurrent processing on maintenance, the Brown-Peterson task is best suited to examine encoding, maintenance, and retrieval processes in WM. The original Brown-Peterson task requires participants to encode and retrieve a string of letters with a concurrent task (i.e., counting backward by three) interposed between encoding and subsequent retrieval [29, 30]. In opposition to the immediate serial recall paradigm, the concurrent task in Brown-Peterson paradigm impairs maintenance and thus retrieval of the encoded information. Here, we designed a novel task inspired from the Brown-Peterson paradigm in which children and adolescents had to maintain verbal information (letters) while performing a concurrent task involving either verbal (lexical decision) or visual (face decision) task appropriate for children and adolescents. This design allowed us to compare not only encoding and retrieval components of verbal WM during fMRI, but also to probe for neural substrates differentially modulated by the concurrent task, both within-domain (i.e. verbal distractors) and cross-domain (i.e. visual distractors). According to the influential model of Baddeley (1986; [31]), verbal and visuo-spatial maintenance and processing involve separate and domain-specific systems, a phonological loop for verbal information and a visuospatial sketchpad for visuospatial information. Thus, processing irrelevant verbal information should produce selective interference with verbal maintenance because verbal processing would mobilize the phonological loop, thus impeding the articulatory rehearsal process in charge of verbal maintenance. By contrast, processing visuospatial information should involve the domain-specific visuospatial sketchpad and should not have any effect on verbal maintenance.

To validate this novel paradigm, we applied it in children and adolescents aged 8 to 16 years. We expected that all would successfully complete our adapted Brown-Peterson fMRI paradigm, which tailors task difficulty to each participant according to their WM capacity. We predicted that distinct activation patterns would be elicited by the two concurrent tasks (i.e. within and cross-domain), not only during the maintenance interval, but also during the subsequent retrieval period. Based on Baddeley’s WM model [30], the nature of the concurrent task was expected to differentially impact verbal WM and thus modulate brain areas recruited during retrieval, despite the fact that identical verbal stimuli were encoded. Specifically, exposure to words vs faces during the maintenance interval should hamper vs favour the engagement of language-related regions in the left hemisphere during the subsequent retrieval phase.

## Materials and methods

### Participants

Participants were 16 healthy children and adolescents aged 8 to 16 years (8 to 10 year-old, n = 5; 11 to 13 year-old, n = 8; 14 to 16 year old, n = 3; mean age = 12.19; SD = 2.25), 9 females and 7 males, recruited though advertisements in local schools and staff at the Royal Children’s Hospital. The wide age range of this sample allowed us to examine whether the adapted Brown-Peterson task was suitable for both children and adolescents. No participant had a documented history of a brain lesion, neurological disability or neurodevelopmental disorder such as autism spectrum disorder (ASD) or attention deficit hyperactivity disorder (ADHD). All participants were right-handed as measured by a score between +40 and +100 at the Edinburgh Handedness Inventory [32, 33], English speaking, had a Full Scale Intellectual Quotient (FSIQ) based on the Wechsler Abbreviated Scale of Intelligence (WASI; [34]) higher than 85 (*M* = 116.2, *SD* = 10.4) and normal or corrected-to-normal vision and hearing. The study was approved by the Human Research Ethics Committee at the Royal Children’s Hospital. Written informed consent was obtained from the caregivers of the children and adolescents prior to participation.

### Material and design

**Fig 1:**
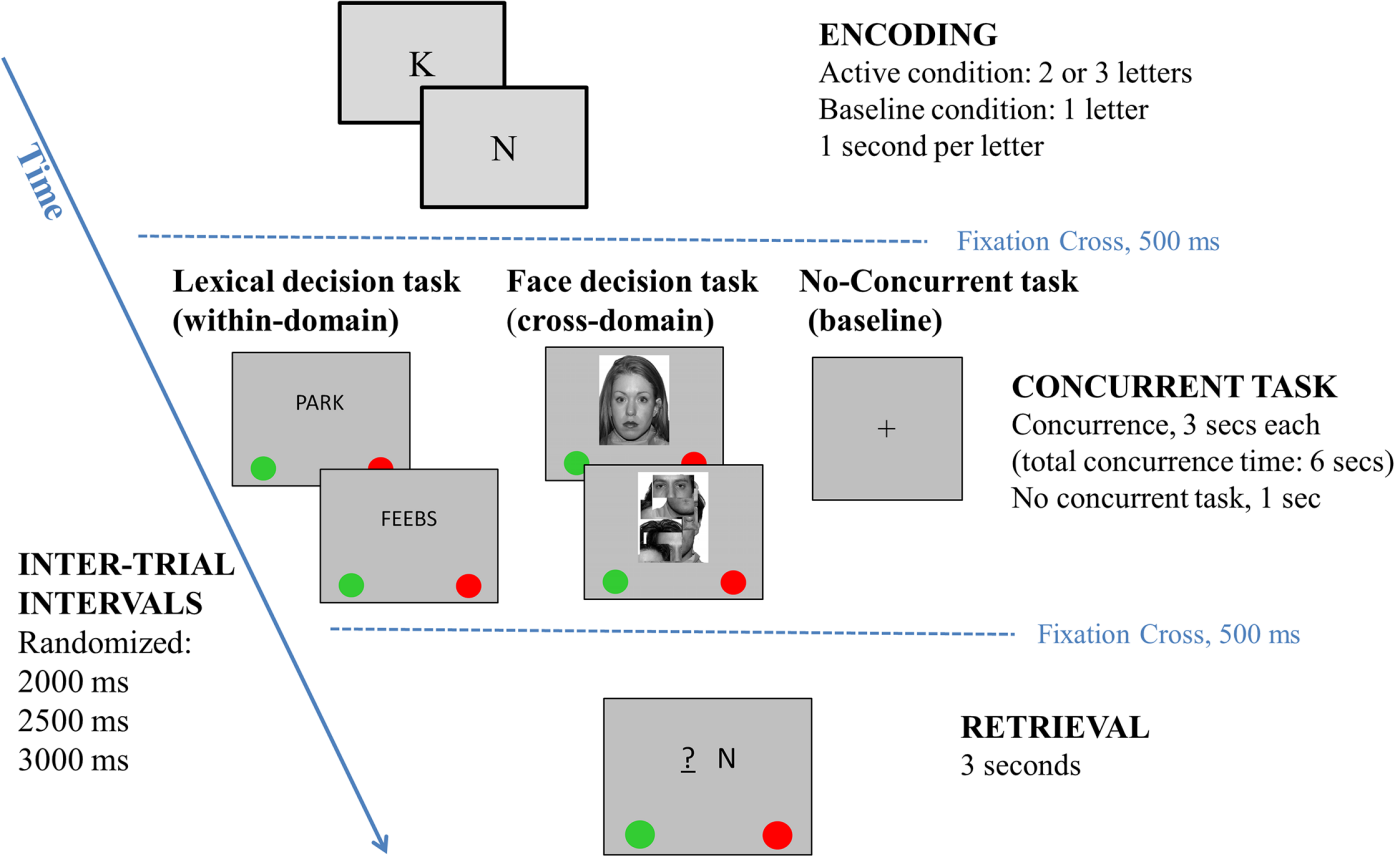
Adapted Brown-Peterson fMRI paradigm using within- and cross-domain concurrent tasks.

Participants completed an adapted version of the Brown-Peterson paradigm [29, 30] implement during functional magnetic resonance imaging (fMRI). A mixed block and event-related design allowed separate examination of specific WM processes: encoding, maintenance and retrieval. The task required a combination of verbal storage and maintenance during either verbal (within-domain) or visual (cross-domain) concurrent tasks. Each active trial consisted of three active phases (Fig 1):

*1)* Encoding period.

Participants were presented with a series of single upper-case letters for further recall displayed sequentially in the middle of the screen at a rate of one letter per second. All consonants of the English alphabet were used as memory items except W, which is three-syllabic. Series of two and three letters were created for within-domain and cross-domain blocks in such a way that each letter appeared with the same frequency in both blocks. Participants were asked to maintain the letters in order of appearance.

*2)* Maintenance delay filled with a concurrent task.

During the maintenance delay of 6 seconds, a concurrent task required to process either verbal or non-verbal stimuli involving within- or cross-domain interference respectively.

The within-domain concurrent task was a lexical decision task. Two successive letter-strings were presented for 3 seconds each and required simple motor responses (i.e. press as quickly and as accurately as possible the left-most/green button if the letter-string was a word; or the right-most/red one if it was a non-word). Words were selected from the “Oxford Wordlist”, which is an Australian database of high frequency words in young children’s writing and reading development [35]. Among the 307 most frequently used words, only nouns were selected based on the following search terms: any gender, any location (urban or rural), any socioeconomical status, any text type (e.g., description, discussion, narrative) and appearing during the first three years of school (40% were within 1 to 100 most frequently used words; 35% were within 101 to 200 most frequently used words; 25% were within 201–307 most frequently used words). Non-words with orthographically existing onsets and bodies were selected from the “ACR Nonword database” [36]. Three to eight letter-strings (words and non-words) were displayed centrally on the screen. Words and non-words were equally often presented.

The cross-domain concurrent task was a face decision task. Two successive pictures were presented for 3 seconds each, requiring similar motor responses (i.e. press as quickly and as accurately as possible the left-most/green button if a real face was presented; or the right-most/red one if it was a scrambled face). Ten males and 10 females faces with a neutral expression were selected from the NimStim database [37], and converted into greyscale using Matlab R2013a (The MathWorks, 2012). Scrambled faces were created from the original faces using Matlab (size of square = 300, iterations = 2). Faces and scrambled faces were equally often presented.

*3)* Retrieval period.

At retrieval, one single upper-case letter was presented along with either one or two place-holders (for paradigm with 2 or 3 letters to remember, respectively) made of dashes with a question mark. Participants had to decide if the single letter matched the letter that was presented in that serial position during the encoding period by giving a simple motor responses, i.e. press as quickly and as accurately as possible the left-most/green button or the right-most/red one for positive and negative responses respectively. This was done to make sure that participants memorised both item and serial order information.

In addition to the active condition, there was a baseline condition (no-concurrent task) in which participants were required to encode a single letter and recognise it after a short empty delay of 1 second. They were instructed to press as quickly and as accurately as possible the left-most/green button if the single letter was the same during encoding and retrieval; or the right-most/red one if it was a different letter.

For both the active and baseline conditions, a randomized inter-trial interval of 2000, 2500, or 3000 milliseconds was presented before the next trial. Three types of blocks of 10 trials each were created: two active blocks, one including the within-domain concurrent task and the other including the cross-domain concurrent task, and a third baseline block. The order of presentation of these three blocks was counterbalanced across participants and repeated twice for a total of six blocks of 10 trials. Within each block, half of the probes were positive (i.e., 5 trials required a “yes” response) and the position of positive and negative probes were randomized within each blocks.

Two important challenges of brain imaging studies examining cognitive development are that differences in both participant age and task performance may influence activation patterns. One concern is whether changes in neural activity reflect changes in functional maturation of the central nervous system, independently of behavioural efficiency, or whether they reflect changes in task performance consequent upon increasing age [22, 38]. For these reasons, in our paradigm, task difficulty was adapted to each participant by adapting the number of verbal items to remember. Based on pilot testing conducted outside the scanner, participants with a backward digit span of 5 or more were presented with the version of the paradigm with 3 letters to be remembered, and those with a backward digit span lower than 5 were presented with the version of the paradigm with 2 letters to be remember. In our sample, seven participants completed the 3-letters paradigm (age range = 10 to 15 years; M = 12.53; SD = 1.44) and nine participants completed the 2-letters paradigm (age range = 8 to 16 years; M = 11.93; SD = 2.78). All participants had a retrieval accuracy of 80% or more, which suggested that task difficulty was appropriate for each participant.

### Procedure

Participants completed the adapted Brown-Peterson fMRI paradigm. This fMRI paradigm was presented visually during fMRI using E-prime2 (Psychology Software Tools, PST, Pittsburgh). Initially, participants successfully completed a mock MRI scanner training protocol before the MRI. Participants were prepared for the adapted Brown-Peterson paradigm through training initially outside (5 trials for each of the three conditions described above) and then inside the scanner before starting fMRI acquisition (again 5 new trials for each of the three conditions). All participants demonstrated understanding of the paradigm before being placed in the scanner. The paradigm was projected onto a screen at the foot of the MRI bed, and participants viewed the images from a mirror attached to the head coil. To minimize head motion during scanning, a soft cloth was placed on the child's forehead, then taped to the head tray, and foam pads were inserted around the head. Responses were provided using an MRI compatible response box with four response buttons. The response box was placed centrally on the child’s stomach and responses were provided by pressing the left-most/green button with the left thumb or the right-most/red button with the right thumb, respectively.

### Statistical analysis of behavioural data on concurrent task and retrieval

Separate repeated measures analyses of variance (ANOVA) were conducted on accuracy measures (percent correct) for the concurrent tasks (within domain/lexical decision task and cross-domain/face decision task) and the retrieval period with the type of the previous concurrent task (within- or cross-domain) as within-subject factor. Independent-sample t tests were used to explore sex differences in accuracy. Pearson’s correlation was used to study the relationship between age and accuracy. Statistical analyses were performed using SPSS Statistics V22.0 [39].

### Image acquisition

MRI was performed on a Siemens 3T MAGNETOM Trio scanner (Siemens, Erlangen, Germany) at the Royal Children’s Hospital. The scanner was equipped with the Syngo MR B17 software release, and a 12-channel receive-only head coil was used. T1-weighted MP-RAGE sequence (Magnetization Prepared Rapid Gradient Echo) were obtained using the following parameters: repetition time (TR) = 1900 ms, echo time (TE) = 2.71 ms, inversion time (TI) = 900 ms, flip angle (FA) = 9°, field of view (FoV) = 256mm, voxel size = 0.7 x 0.7 x 0.7 mm. Functional images were acquired using a T2-weighted with a gradient-echo-planar imaging (EPI) sequence with 32 interleaved slices with 5% gap, voxel size = 2.6 x 2.6 x 3 mm, TR = 2400ms, TE = 35ms, FA = 90°, FoV = 240mm.

### Image analysis

fMRI data were preprocessed and analysed using SPM8 (Wellcome Department of Imaging Neuroscience, University College London, UK) implemented in Matlab R2014a. The images of each subject were corrected for slice acquisition timing, and spatially realigned to eliminate movement artefacts. Head motions were small in any direction (Maximum translation, X = 0.39mm, Y = 0.76mm, Z = 1.69mm; Maximum rotation (converted from degrees to millimetres, 40): X = 0.04mm, Y = 0.2mm, Z = 0.01mm; Mean translation: X = 0.08mm, Y = 0.11mm, Z = 0.25mm ; Mean rotation : X = 0.004mm, Y = 0.003mm, Z = 0.002mm) and therefore no participant was excluded from further processing [40]. To allow for inter-subject comparison, data were normalized using the MNI brain template (Montreal Neurologic Institute) and resampled to 1.9 x 1.9 x 3 mm. These functional images were finally smoothed using a Gaussian filter of full width at half maximum = 8mm to increase signal-to-noise ratio.

Statistical analyses were performed using a two-step process, taking into account the intra-individual and inter-individual variance [41]. First level single subject statistics were assessed by a voxel-based statistics according to the General Linear Model implemented in SPM8. Given the high rate of correct responses across participants (above 90%, see Results section for further detail) and to guarantee an equal number of trials for each condition, brain activity was analysed pooling the correct and incorrect trials together. The onsets of each event of interest were convolved with the canonical hemodynamic response function (HRF) and used as regressors in the individual design matrix. For the encoding period, these onsets included encoding of the active condition and encoding of the baseline condition, using a boxcar function of 2 or 3 seconds for active encoding (depending of the difficulty level) and 1 second for the baseline encoding. The maintenance delay filled with a concurrent task was modelled using a boxcar function of 6 seconds for the within-domain (lexical decision) and the cross-domain (face decision) concurrent tasks. Finally, the retrieval period was modelled using a boxcar function of 3 seconds for the tree retrieval types, i.e., retrieval after within-domain concurrent task, retrieval after cross-domain concurrent task and retrieval of the baseline condition.

All six movement parameters (translation: x, y and z; rotation: pitch, roll and yaw) were included as covariates of no interest in the model. The individual statistical images from each condition were then entered in a group analysis at the second level using a flexible factorial design, which provides the flexibility to specify the different period of our mixed block and event-related paradigm. In this random-effects model, independence and unequal variance between subjects and conditions were assumed, allowing for violation of sphericity, as implemented in SPM8. Considering a possible impact of gender on brain-activation, we also added this binary variable as a covariate in the flexible factorial design [26, 38, 42, 43]. In line with guidelines used in neuroimaging studies of complex cognitive functions [44], whole-brain analysis was conducted with a significance threshold of p < .001 at the voxel level, uncorrected for multiple comparisons, and a minimum extent threshold of 20 voxels [26, 45]. Anatomical location of activations was verified using SPM Anatomy toolbox [46].

We performed exploratory analyses to examine age- and retrieval accuracy-related changes in brain activation during the Brown-Peterson fMRI paradigm. The largest and most relevant clusters of activation identified at the group level were used to define functional regions of interest (ROIs) for each of the different conditions using the marsBaR toolbox [47]. Beta values were extracted from each ROI, by contrasting activation during the encoding or retrieval WM conditions relative to the respective baseline conditions. Beta values from each ROI and each participant were then used to compute Pearson’s correlation coefficients in order to evaluate any age- and accuracy-related effects on ROI activity using SPSS [39]. Beta values from the encoding or retrieval periods were contrasted to the baseline values (rather than to each other) to test for condition-specific effects without mixing any positive vs negative correlation with one vs the other active condition.

We also performed a whole-brain analysis where different active phases were compared (encoding vs retrieval, within-domain concurrent task vs cross-domain concurrent task, retrieval following within-domain concurrent task vs retrieval following cross-domain concurrent task), but now including age and retrieval accuracy as covariates of interest in a multiple parametric regression design using SPM8. For these regressions, a significant threshold of p < .001 uncorrected for multiple comparisons with a minimum extent threshold of 20 voxels was used.

## Results

### Behavioural data

As far as the concurrent tasks were concerned, the percentage of correct responses was 97% (SD = 4.3) for the within-domain (lexical decision task) and 98% (SD = 3.5) for the cross-domain (face decision task). For the effect of the type of the concurrent task, assumption of normality was violated, as assessed by inspection of histograms and results of the Shapiro-Wilk test (p = .001). Therefore, related-sample Wilcoxon-signed rank test was used and showed no significant effect of the type of concurrent task (W_s_ = 33, z = .58, p = .565). Concerning retrieval of the active condition, repeated-measures ANOVA showed no effect of type of concurrent task on response accuracy, *F* (1,15) = 1.278, *p* = .276 (90.9%, SD = 8.8, and 93.4%, SD = 5.3, for the within-domain/lexical and cross-domain/face decision tasks, respectively). Hence, differences in brain activity patterns at retrieval could not be explained by differences in WM performance.

There was no significant relationship between age and response accuracy on the retrieval of the active condition whatever the type of the previous concurrent task (*r* = .318, *p* = .23, and *r* = .299, *p* = .261 for the within- and between-domain concurrent task respectively), and no significant relationship between age and response accuracy on the concurrent tasks (*r* = .493, *p* = .052, and *r* = .185, *p* = .492 for the lexical decision and face decision concurrent tasks, respectively). There was no significant gender difference for any of the measures, *t*s < 1, *p*s > .50.

Taken together, these behavioural data show good performance overall on the adapted Brown-Peterson paradigm. Moreover, this pattern was stable across the age range of our sample and gender. Therefore, from a behavioural point of view, our task appears to be suitable for a wide age range of children and adolescents.

### Functional magnetic resonance imaging

#### Active letter encoding and retrieval vs. baseline

To delineate brain regions generally recruited during WM, we first contrasted the active encoding period relative to the baseline encoding period, regardless of the domain of concurrent task during the maintenance interval. This showed activation in a widespread network, including bilateral visual areas in the occipital lobes, parahippocampal gyri, as well as left prefrontal regions, the caudate nucleus, and the cerebellum (Table 1). Likewise, we contrasted the active retrieval relative to the baseline retrieval period, regardless of concurrent conditions, which revealed a distributed pattern of activation encompassing mainly bilateral prefrontal cortices, but also temporal and parietal areas (Table 1). These data confirm that our working memory paradigm successfully engaged brain networks associated with visual stimulus processing and executive functions.

**Table 1:** List of activations for active encoding and retrieval compared to baseline condition.

Region		Hemisphere	Number of voxels	t value	x, y, z
**ENCODING (compared to encoding baseline)**					
*Frontal*	Inferior (BA 47)	L	108*	4.46	-38, 30, -14
	Superior and middle (BA 9)	L	160*	4.42	-27, 40, 43
	Superior and superior medial (BA10)	L	193*	4.22	-15, 57, 13
*Occipital*	Lingual, inferior, calcarine (BA18)	L	515*+	6.33	-25, -95, -11
				4.66	-11, -99, -8
		R	631*+	6.08	25, -91, -11
				5.89	21, -91, -2
*Temporal*	Parahippocampal gyrus	L	130*+	5.07	-40, -28, -11
		R	71*	4.77	13, -13, -17
*Subcortical*	Caudate nucleus (BA 48)	L	563*+	5.87	-17, 19, 10
	Pulvinar	R	24	3.64	13, -32, 13
	Cerebellum	L	222*	4.77	-10, -30, -14
**RETRIEVAL (compared to retrieval baseline)**					
*Frontal*	Prefrontal, putamen, middle and inferior (BA 49, 10, 44)	L	7684*+	6.10	-15, -6, 13
				6.07	-27, 8, -2
				5.83	-29, 42 19
				5.05	-61, 11, 22
	Middle and superior (BA 10, 6)	R	572*+	4.91	27, 46, 7
				4.43	28, 51, 10
			23	4.01	36, -2, 64
	Superior orbital (BA 11)	L	74*	4.77	-21, 53, -14
	Precentral gyrus (BA 6, 4)	L	268*	5.13	-34, -4, 61
		L	56	4.18	-49, 0, 40
		L	32	3.49	-36, -17, 40
	Middle cingulate (BA 24)	L	42	3.82	-17, -25, 46
*Parietal*	Angular (BA 39)	R	169	4.21	40, -65, 46
	Inferior and superior lobule (BA 7)	L	1813*+	5.02	-36, -55, 55
				4.92	-32, -61, 55
	Inferior lobule and postcentral gyrus (BA 40, 1)	L	404*+	4.85	-51, -25, 46
				4.19	-57, -23, 28
*Temporal*	Middle extending calcarine gyrus	R	189*+	5.74	32, -65, 16
	(BA23)			3.49	28, -57, 10
	Superior and middle (BA 39)	L	82	4.25	-61, -47, 19
	Middle (BA 21)	R	58	3.83	51, -34, -14
*Occipital*	Lingual (BA 18)	L	214	3.98	-6, -76, -2
*Subcortical*	Vermis	L	229	4.68	-2, -53, -5
	Cerebellum	L	156	4.32	-25, -61, -17

Note: Coordinates are in MNI space. x, y, z coordinates refer to voxels with highest statistical significance within a cluster (location of the peak coordinate).

Clusters used to define ROIs for specific subsequent analyses are marked with a sign *.

Clusters reaching a significance threshold of p < .05 at the voxel level, corrected for multiple comparison, are marked with a sign +. BA = Brodmann area

#### Active letter encoding vs. letter retrieval

**Fig 2:**
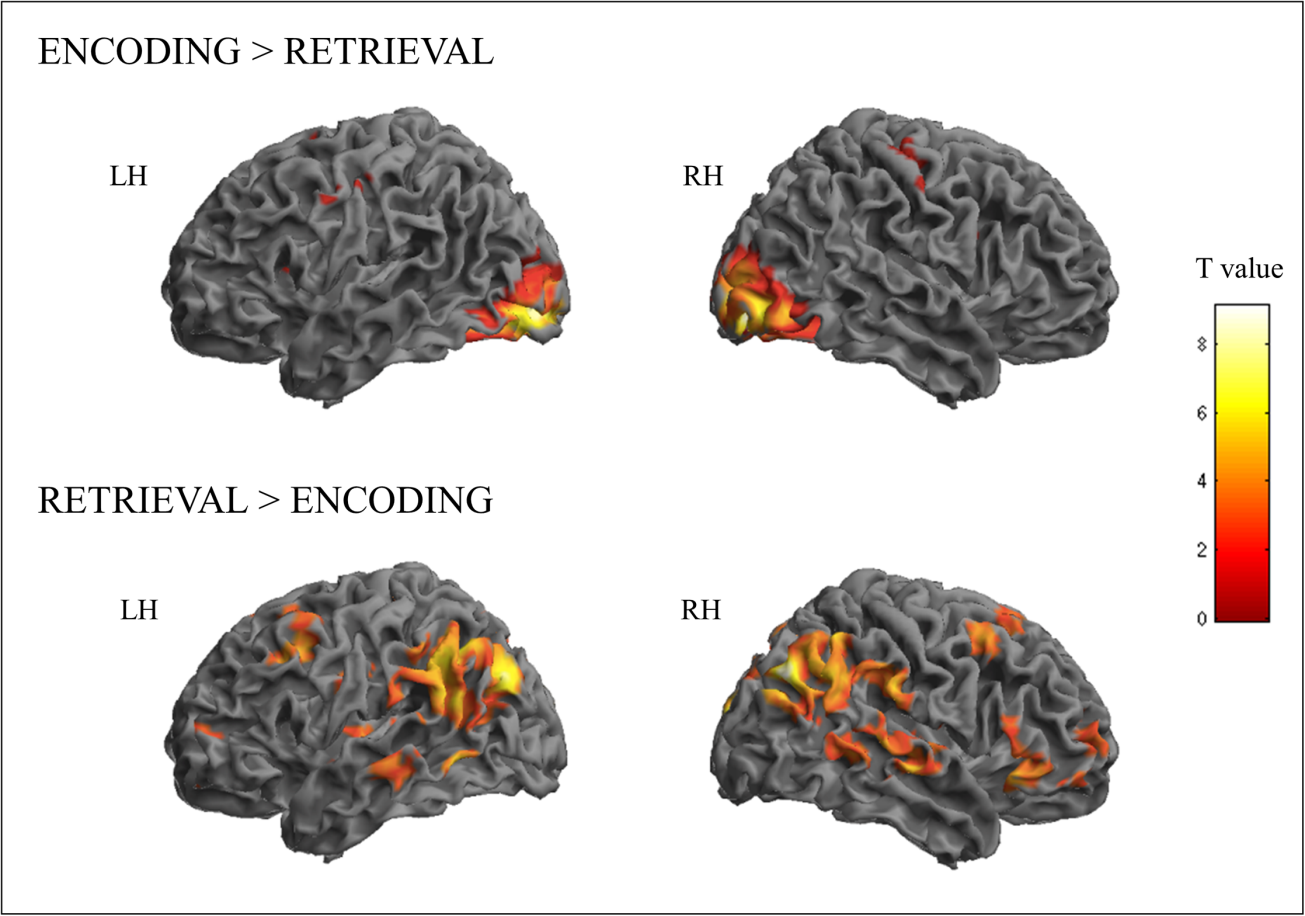
Activation maps related to the contrasts encoding vs retrieval.

We next sought to identify regions selectively recruited by distinct WM processes. Encoding, as compared to retrieval (during the active task), was associated with widespread activations bilaterally in the occipital and ventral temporal lobes (inferior occipital and fusiform gyri), as well as in medial frontal areas (supplementary motor area (SMA), middle cingulate gyrus) and precentral gyrus. Smaller activation foci were found in the insula (Fig 2 and Table 2). Conversely, the retrieval phase, compared to encoding, activated bilateral dorsolateral prefrontal areas (mainly inferior and middle, but also superior frontal gyri), as well as the anterior cingulate cortex (ACC), inferior parietal lobule (angular, supramarginal, and postcentral gyri), and lateral temporal areas (superior and middle temporal gyri).

**Table 2:** List of activations for contrasts of interest.

Region		Hemisphere	Number of voxels	t value	x, y, z
**ENCODING > RETRIEVAL**					
*Frontal*	SMA, middle cingulate (BA 6)	L&R	640*+	6.84	-6, 8, 49
				5.45	8,10,49
	Pre and post central gyrus (BA 4)	L	175	4.07	38, -21, 55
	Precentral gyrus (BA 6)	R	104+	5.32	46, 6, 28
	Medial (BA 11)	L	23	3.68	-0, 38, -17
*Parietal*	Postcentral gyrus (BA 6, 1)	L	70+	5.09	-53, -6, 49
		R	25	4.05	61, -13, 46
*Occipital*	Inferior (cuneus, precuneus, lingual), fusiform (BA 18, 19, 37)	L	2468*+	13.24	-23, -89, -11
				11.65	-36, -80, -11
				8.94	-36, -51, -17
		R	2692*+	13.11	27, -87, -11
				8.28	34, -49, -17
				8.25	32, -89, 10
*Other*	Insula (BA 13)	L	31	3.63	-30, 13, 10
**RETRIEVAL > ENCODING**					
*Frontal*	Precentral, middle (BA 8, 6) Middle (BA 8, 10)	L	500*	4.87	-36, 11, 40
				4.78	-38, 13, 37
		R	227*	4.6	40, 10, 49
		L	59	3.64	-44, 51, 10
	Inferior, middle (BA 47, 10)	R	487*	4.68	47, 23, -8
				4.27	44, 53, -11
	Superior and middle (BA 10)	R	212	4.2	30, 63, 4
	Superior, SMA (BA 8, 6)	R	242*	4.1	25, 23, 55
				4.08	9, 25, 58
	Anterior cingulate (BA 32)	R	227*	4.09	2, 36, 19
	Precentral gyrus (BA 4)	L	34	4.04	-19, -27, 55
	Middle orbital (BA 10)	L	34	3.93	-29, 57, -11
	Superior medial (BA 8)	R	24	3.57	2, 34, 40
*Parieto-temporal*	Angular, superior temporal, supramarginal, inferior parietal lobule (BA 39, 22)	R	3563*	7.3	46, -74, 34
				5.17	59, -19, -5
				4.94	46, -53, 49
	Angular, middle temporal, inferior parietal lobule	L	5998*	6.41	-42, -55, 40
				6.02	-55, -51, 22
				5.35	-49, -51, 37
	Postcentral gyrus (BA 4)	L	188*	4.91	-42, -13, 31
*Temporal*	Superior extending to putamen (BA 49)	L	301	4.7	-30, -13, 4
	Middle (BA 21)	L	190	4.18	-65, -25, -8
*Occipital*	Lingual (BA 18)	R	25	3.8	11, -74, -8
*Subcortical*	Putamen (BA 49)	R	199	4.44	30, -13, 7
**WITHIN-DOMAIN > CROSS-DOMAIN CONCURRENT TASK**					
*Frontal*	Frontal pole (BA 10)	R	266*+	5.3	27, 55, 4
*Occipital*	Medial fusiform (BA 19)	R	36*	4.43	30, -53, -8
**CROSS-DOMAIN > WITHIN-DOMAIN CONCURRENT TASK**					
*Occipital*	Inferior (lingual, precuneus, fusiform), cuneus, including fusiform face area (FFA; BA 19, 18, 37)	R	2873*+	9.1	42, -84, -11
				8.97	34, -91, -5
				5.92	49, -53, -14
	Middle, lingual, inferior, lateral fusiform, including FFA (BA 19, 18, 37)	L	878*+	5.64	-34, -91, -5
				4.76	-44, -72, -14
				4.6	-48, -51, -17
	Precuneus gyrus (BA 7)	R	30	3.79	8, -59, 64
	Lingual (BA 18)	L	39	3.79	-0, -61, 7
*Frontal*	Inferior (BA 47)	L	238*+	5.11	-38, 36, -14
	Precentral (BA 4)	R	156+	4.9	38, -13, 43
	Medial frontal (BA 11)	L	92	4.79	-2, 46, -17
	Middle cingulate (BA 24)	R	92	4.22	13, -17, 49
	SMA (BA 6)	L	59	3.82	-6, -13, 55
*Temporal*	Inferior (BA 20)	R	39*+	5.29	47, -27, -20
	Middle (BA 21)	L	60	4.12	-61, -9, -20
	Parahippocampal gyrus	L	806*+	5.63	-29, -11, -14
*Parietal*	Inferior lobule (BA 40)	R	119+	5.06	57, -27, 55
	Postcentral gyrus (BA 4)	L	92	4.32	-42, -27, 64
	Angular (BA 39)	L	169	4.1	-36, -59, 22
	Superior lobule (BA 7)	R	59	4.06	25, -70, 52
*Subcortical*	Pulvinar	R	207*+	5.28	25, -30, 7
**RETRIEVAL AFTER WITHIN-DOMAIN > RETRIEVAL AFTER CROSS-DOMAIN CONCURRENT TASK**					
*Occipital*	Cuneus, fusiform, middle and inferior occipital (BA 18, 19)	R	3181*+	8.71	15, -101, 7
				8.44	27, -78, -8
				7.58	30, -89, 10
				7.17	42, -72, -8
	Inferior and middle occipital, fusiform, calcarine (BA 18, 37)	L	1620*+	7.15	-25, -80, -8
				6.71	-32, -61, -14
				6.62	-15, -101, 4
				5.47	-6, -91, -11
**RETRIEVAL AFTER CROSS-DOMAIN INTERFERENCE > RETRIEVAL AFTER WITHIN-DOMAIN CONCURRENT TASK**					
*Temporal*	Middle and superior (BA 21)	L	27*	3.74	-40, -47, 4
		L	23*	3.39	-59, -34, 4
*Occipital*	Calcarine (BA 17)	R	279*	4.79	2, -91, 10
	Inferior (BA 37)	L	22*	3.83	-53, -63, -14

Note: Coordinates are in MNI space. x, y, z coordinates refer to voxels with highest statistical significance within a cluster (location of the peak coordinate).

Clusters used to define ROIs for specific subsequent analyses are marked with a sign *.

Clusters reaching a significance threshold of p < .05 at the voxel level, corrected for multiple comparison, are marked with a sign +. BA = Brodmann area.

#### Maintenance delay filled with a concurrent task (within-domain vs. cross-domain)

**Fig 3:**
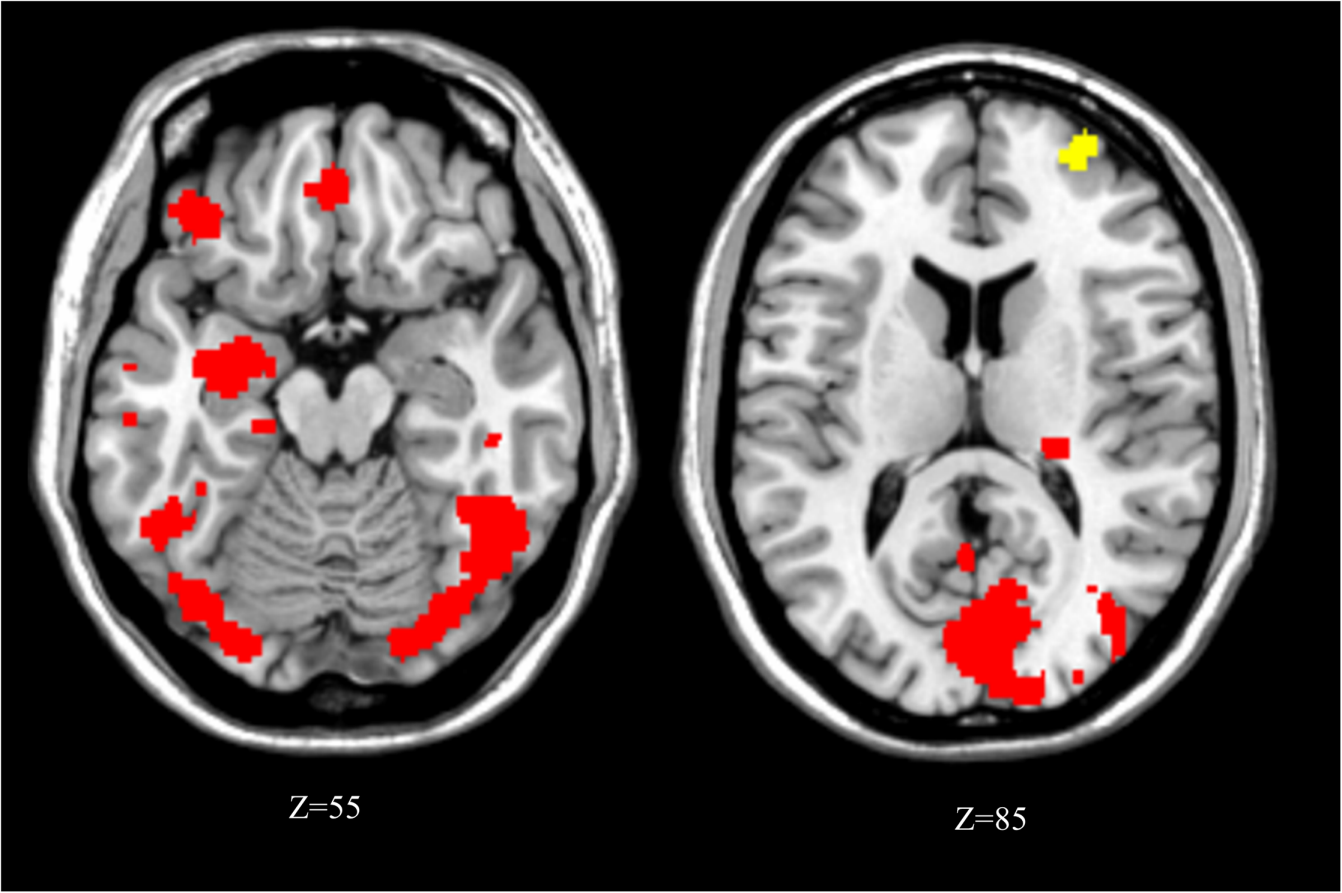
Activation map for the contrast within-domain vs cross-domain concurrent tasks (MRIcron reference slices). Activations in yellow: within-domain concurrent task > cross-domain. Activations in red: cross-domain concurrent task > within-domain.

Comparing activations during the within-domain concurrent task (lexical decision task), relative to the cross-domain concurrent task (face decision task), revealed differential increases in the right middle frontal gyrus (Brodmann area 10) and medial fusiform cortex only (Table 2 and Fig 3). Conversely, the cross-domain concurrent task (face decision task) compared to within-domain concurrent task (lexical decision task) produced a more extensive pattern of activation, particularly in bilateral visual areas, including occipital and fusiform cortex overlapping with the fusiform face areas (FFA). Activations were also found in several frontal areas (left inferior and medial frontal gyri, SMA, right middle cingulate cortex, precentral gyrus), the temporo-parietal junction, left parahippocampal gyrus, and right pulvinar. Thus, the cross-domain concurrent task appeared to recruit a more widespread network than the within-domain concurrent task, even though behavioural data show that this could not be explained by task difficulty since accuracy did not significantly differ in the two concurrent tasks.

#### Letter retrieval following within-domain vs. cross-domain concurrent tasks

**Fig 4:**
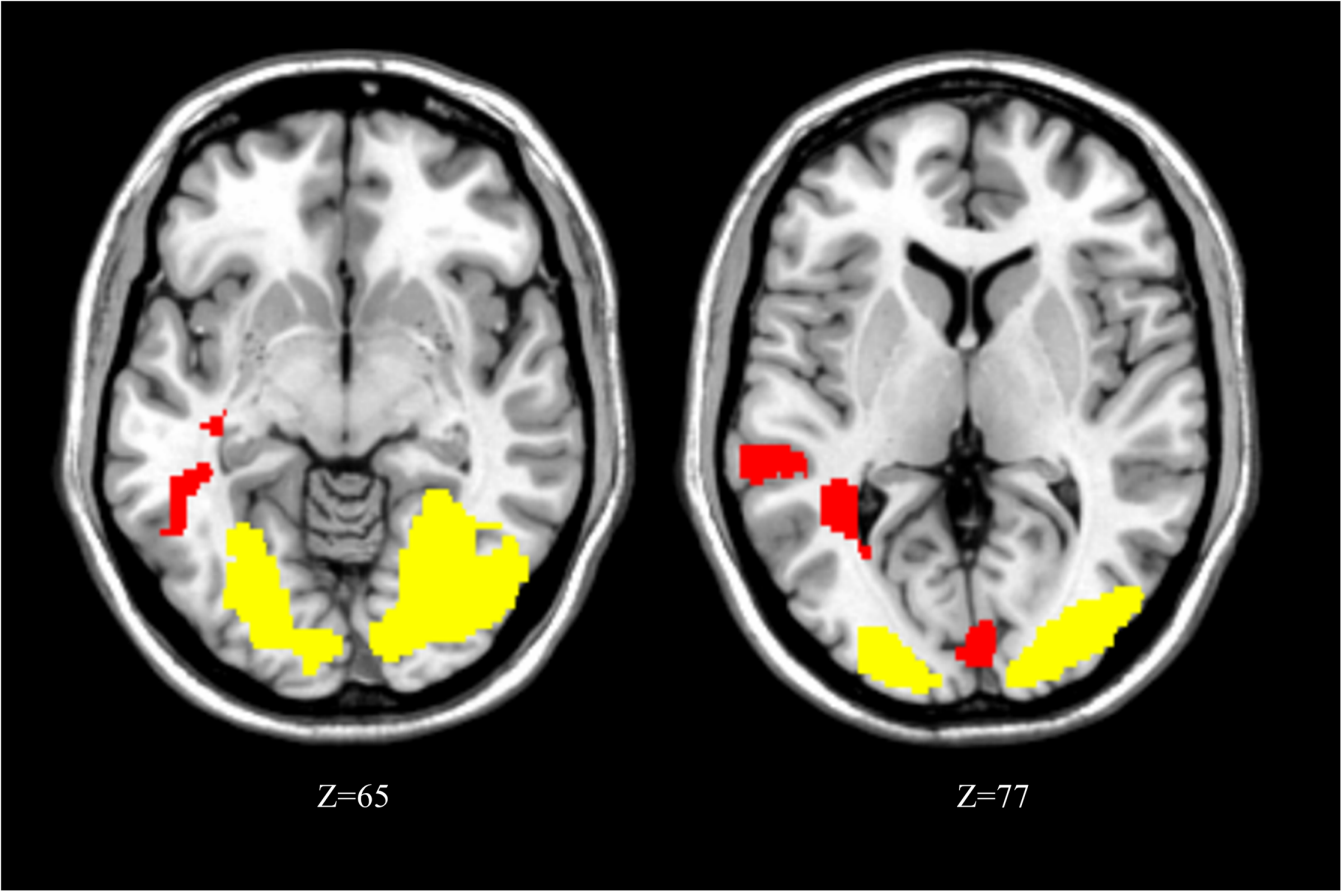
Activation map for retrieval following within-domain vs cross-domain (MRIcron reference slices). Activations in yellow: retrieval following within-domain concurrent task > cross-domain. Activations in red: retrieval following cross-domain concurrent task > within-domain. For illustration purpose, activations observed in retrieval following within-domain concurrent > cross-domain are represented with a threshold of p < .005 uncorrected for multiple comparisons.

The most critical question concerning the WM system in our paradigm is whether the nature of the concurrent task during the maintenance interval may produce different degrees of competition and thus result in different neural substrates during retrieval. We therefore tested for brain regions that would be differentially activated during the retrieval period when following within-domain concurrent task (lexical decision) or when following cross-domain concurrent task (face decision). Greater increases following the within-domain concurrent task were found in visual areas, with large bilateral clusters in occipital cortices (bilateral middle and inferior occipital gyri, fusiform gyri, right cuneus and left calcarine). Conversely, greater increases were found after the cross-domain concurrent task in the left middle and superior temporal cortex, overlapping with usual location of phonological processing [48, 49], plus left calcarine gyrus and bilateral medial occipital cortex (Table 2 and Fig 4).

#### Age and retrieval accuracy-related activations

Several functional ROIs were defined for each of the contrast of interest described above (marked with a star in Tables 1 and 2) and used for additional analyses to examine any modulation by individual characteristics of the participants. Parameter estimates (beta values) extracted and averaged across voxels from these ROIs were then submitted to Pearson’s correlation with age and WM retrieval accuracy. No significant correlation was found between encoding- or retrieval-related activation (relative to baseline activation) with neither age nor WM retrieval accuracy on the adapted Brown-Peterson paradigm for any of these ROIs. Table 3 summarizes these correlation coefficients.

**Table 3:** Pearson’s correlations between activity of functional ROIs and (a) age or (b) retrieval accuracy.

Functional ROIs				Age		Accuracy	
Region	Side	Number of voxels	Peak coordinates	r	P value	r	P value
**ENCODING (compared to encoding baseline condition)**							
*Frontal* Inferior	L	108	-38, 30, -14	-.351	.183	-.275	.304
Superior and middle	L	160	-27, 40, 43	-.041	.881	-.269	.314
Superior and superior medial	L	193	-15, 57, 13	-.101	.711	-.360	.171
*Occipital* Lingual, inferior, Fusiform	L	515	-25, -95, -11	.322	.224	.235	.382
	R	631	25, -91, -11	.308	.245	.182	.501
*Temporal* Parahippocampal Gyrus	L	130	-40, -28, -11	.455	.077	.214	.426
	R	71	13, -13, -17	.225	.401	.389	.137
*Subcortex* Caudate Nucleus	L	563	-17, 19, 10	-.225	.402	-.111	.683
*Other* Cerebellum	L	222	-10, -30, -14	.241	.369	.361	.169
**RETRIEVAL (compared to retrieval baseline condition)**							
*Frontal* Inferior extending to putamen and insula	L	7684	-15, -6, 13	-.174	.519	-.100	.171
Middle and superior	R	572	27, 46, 7	-.010	.969	.055	.839
Precentral	L	268	-34, -4, 61	.242	.367	.282	.289
Superior orbital	L	74	-21, 53, -14	.030	.911	.076	.780
*Temporal* Middle extending to precuneus	R	189	32, -65, 16	.187	.489	.405	.120
*Parietal* Inferior and superior lobule	L	1813	-36, -55, 55	.395	.130	.248	.354
Inferior lobule, postcentral	L	404	-51, -25, 46	.338	.201	.381	.145

Note: Activity was measured during either encoding or retrieval periods depending on the phases recruiting each ROI. Coordinates in MNI space and number of voxels are given for each functional ROI, as well as Pearson’s correlation coefficients, r, and corresponding p values.

We also performed an exploratory whole-brain regression analysis in SPM using (a) age; and (b) WM retrieval accuracy for the main contrasts of interest as described above (encoding vs retrieval, within vs cross domain concurrent tasks). None of these analyses revealed any significant overlap with activations identified by the main contrasts of interest reported in Table 2 indicating that all effects reported above are largely independent of age (within the range of our sample) and WM retrieval accuracy.

## Discussion

We report and validate an adapted Brown-Peterson fMRI paradigm that probes for the neural correlates of different WM processes, including encoding, maintenance and retrieval, as well as the effect of within- and cross-domain concurrent tasks during maintenance. Results indicate that this paradigm can be performed equally well by children and adolescents of different ages, with reliable results across different levels of performance. To our knowledge, this is the first study to propose a paradigm to delineate distinct patterns of brain activity for the different WM processes in children and adolescents. We provide the first exploratory results on brain activity related to encoding, maintenance, and retrieval WM processes in children and adolescents, and compare verbal WM in the presence of both verbal (within-domain) and visual (cross-domain) concurrent tasks.

As expected, our adapted Brown-Peterson paradigm was successfully completed with high accuracy in the MRI scanner by typically developing children as young as 8 years of age, indicating that it is suitable to examine WM processes in children and adolescents from 8 to 16 years of age. It is important to note that task difficulty was adapted to each participant’s WM capacity using a simple procedure (based on backward digit span performance, the participant completed the paradigm with two or three letters to remember), and we found no significant association between age or task performance and brain activation patterns. These findings indicate that our paradigm is well suited to examine brain systems associated with different WM capacities in different age groups. This may be an important advantage when comparing groups with different developmental trajectories, because previous studies show that WM-related activations may increase with age in parallel with changes in performance and improvements in WM capacity [12, 18].

Secondly, our imaging results demonstrate that, while distributed networks in frontal and visual areas activated in the context of the verbal WM paradigm used here (i.e. during the active conditions compared to the baseline), distinct neural substrates were selectively recruited during the encoding and retrieval periods. The verbal encoding period induced stronger activations in posterior and ventral brain regions, with large bilateral increases in occipital, as well as parahippocampal cortices. In contrast, the verbal retrieval period induced stronger activations in more anterior and dorsal regions, in particular in prefrontal and parietal areas, and to a lesser extent in lateral temporal areas.

The predominance of activity in visual cortex together with medial temporal lobe (parahippocampal gyrus) during encoding is consistent with the need to extract discriminative visual information from the to-be-remembered stimuli and store this information into short-term memory. On one hand, ventral occipito-temporal areas differentially engaged during encoding are crucial for perceptual shape analysis, especially for letters with a letter-sensitive activation in these regions [50, 51]. We did not find selective activations corresponding to the “visual word form area” but this region is typically responsive to letter-strings or words rather than isolated letters [52–54]. Moreover, we did not find language-related activation during verbal encoding, in particular Broca’s area which has been implicated in the subvocal rehearsal system [55]. However, language-related activation has been mainly found during encoding of words [56] and not during encoding of letters [57].On the other hand, the parahippocampal cortex is a key brain region at the interface between perception and memory, therefore likely to make an important contribution to efficient storage of visual information into WM [58].

As expected, predominant activity in frontal and parietal areas during retrieval is consistent with executive control and attentional focusing. The executive control system serves as an attention controller that allocates and coordinates attentional resources for cognitive tasks, such as retrieval of information encoded in working memory [59, 60]. Our findings accord with previous studies showing the involvement of frontal areas, especially prefrontal and anterior cingulate cortices, in the executive control required during WM demands [61, 62]. Focusing attention is crucial for efficient executive control [63] and recruits parietal regions [64], which were strongly implicated during the retrieval period in our study. In addition, WM retrieval of serial order is dissociable from the type of information contained in the item sequence [65] and also relies on activation in frontal and parietal activations [66].

Overall, our findings converge with those of van den Brosh and colleagues (2014), who reported a similar posterior and perceptual network during the encoding phase compared to a more anterior and executive network during the recall phase of a Sternberg item recognition paradigm (which did not include a distracting phase) in children and adolescents aged 9 to 19 years. However, these authors did not find any temporal or parahippocampal activations, possibly reflecting differences in the paradigm and material used (digits in their study vs. letters in ours). More generally, our findings of extensive fronto-parietal and visual activity during WM also dovetail with previous neuroimaging studies investigating brain systems associated with verbal WM in children and adolescents, across different kinds of verbal WM paradigms, such as the Steinberg item recognition task using letters [21, 67, 68] or n-back tasks using letters [69, 70].

Study hypotheses were supported by results revealing that brain activation patterns differ as a function of the nature of the concurrent task performed during the maintenance interval. Our design allowed us to compare the impact of within-domain (lexical decision task) versus cross-domain (face decision task) concurrent task processing during the maintenance period intervening between encoding and retrieval, while information stored in WM itself did not differ. A lexical decision task was expected to produce within-domain interference, as it involved verbal material resembling the to-be-remembered material (i.e. letters), while a face decision task was considered to induce cross-domain interference as it relied on non-verbal visual processes.

As predicted, the within-domain and cross-domain concurrent tasks evoked distinct brain activations when compared to each other. Localised and right-sided activations in the right frontal pole (Brodmann area 10) and medial fusiform gyrus were observed during the within-domain/lexical concurrent task, whereas the cross-domain/face concurrent task elicited much more distributed activations in occipital temporal extrastriate areas, but also left parahippocampal gyrus and fronto-parietal regions. These differences could not be attributed to task difficulty (since there were no significant difference in accuracy between the within-domain/lexical and the cross-domain/face decision task) but most likely reflect the different task demands and perhaps different strategies and processes applied during the maintenance interval. Since verbal information had to be held in WM, it might have produced stronger interference and greater conflict in resource allocation during the within-domain/lexical decision task than the cross-domain/face decision task, eventually affording less efficient engagement of task-specific networks in the former condition and hence lower accuracy. The involvement of the right frontal pole (Brodmann area 10), thought to organize an optimal use of cognitive resources and overcome potential impasses [71], may reflect this conflict in resource allocation and an increase in cognitive load during a verbal concurrent task. Such recruitment of attentional control mechanisms during interference appears consistent with the time-based resource-sharing model (TBRS; [72–74]). This model postulates the existence of attention-based mechanisms involved to maintain relevant verbal information when the capacity of the verbal-specific system (comparable to the phonological loop in Baddeley and Hitch’s model) is exceeded [75]. Alternatively, greater activation of visual and fronto-parietal areas as well as temporal regions, including parahippocampal gyrus, during the cross-domain/face decision task might reflect the dual process of face decision task and active maintenance of verbal information.

Critically, and in keeping with our hypotheses, the two concurrent tasks (within- and cross-domain) elicited distinct patterns of brain activity during the subsequent retrieval phase, despite the fact that identical stimuli were encoded, maintained and retrieved from WM. This indicates that partly different processes mediated retrieval after within- and cross-domain interference, and thus WM retrieval differed according to the nature of the preceding concurrent task. Large bilateral occipital activations were engaged during retrieval after the within-domain/lexical concurrent task, whereas only limited activity was observed in medial occipital cortex in addition to left superior and middle temporal cortex during retrieval after the cross-domain/face concurrent task. Interestingly, the latter cluster in temporal cortex overlapped with regions often reported in phonological tasks and associated with language networks [48, 49]. A plausible explanation for such difference would be that the maintenance of letters relied on a preferentially visual format when a concurrent verbal task had to be performed (i.e., within-domain concurrent task), hindering the use of the phonological loop for maintenance. On the other hand, the visual concurrent task may not prevent maintenance in the phonological loop, explaining a lesser involvement of visual cortex but conversely greater recruitment of language-related areas (left superior and middle temporal) during retrieval. These interpretations would accord with Baddeley and Hitch’s model previously mentioned, and the proposed effect of articulatory suppression on verbal WM [59, 76, 77].

The current study is not without limitations. The study sample size could be considered relatively small. We note, however, that it is comparable with previous studies exploring neural correlates of WM [12, 67, 78]. Even if our data showed no hint of any systematic modulation of brain activity patterns by age or retrieval accuracy, correlation and regression analysis performed here can be sensitive to small size. Nevertheless, by design, our procedure of tailoring task difficulty to each participant according to their WM capacity precisely aimed at avoiding age related effects and minimizing confounding effects due to individual differences in performance. We acknowledge that the lack of variability and the high retrieval accuracy resulting from this procedure may have limited the sensitivity of our study to activations modulated by age or other individual factors. Another limitation is that our paradigm did not test the reverse situation of verbal versus visual concurrent tasks on visual information held in WM. Examining both verbal and visuospatial WM in the presence of verbal and visuospatial interference could map more precisely how the different processes subserving verbal and visuospatial WM are influenced by different kinds of concurrent tasks.

## Conclusions

Our study provides new insights into WM-related brain activity. We show a greater role of perceptual brain systems for encoding processes, and a fronto-parietal attentional network for retrieval processes. More critically, we show that a concurrent task during maintenance in WM produced distinct activations not only during the concurrent task itself, but also during subsequent retrieval. We conclude that the specific demands of the concurrent task affect the way memory items are maintained in WM, selective verbal interference resulting in greater reliance on visual cortex for retrieval, whereas visual interference leaves verbal systems of maintenance unaffected, hence resulting in the involvement of language-related areas in left temporal cortex for retrieval. These data accord with WM models postulating differentiated cognitive processes, with distinct neural substrates, according to the concurrent material interfering in verbal WM [59, 76, 77]. In addition we show that these activation patterns are robust across different ages and different WM capacities. More generally, our work validates a new WM paradigm derived from the Brown-Peterson task allowing us to probe for the neural correlates of different WM processes. Because the difficulty of the task was adapted to each participant and results were stable across age, this fMRI paradigm may be usefully applied in developmental populations with a wide age range and also feasible in clinical paediatric population (e.g., populations with mild intellectual difficulties).
